# A componential view of children's difficulties in learning fractions

**DOI:** 10.3389/fpsyg.2013.00715

**Published:** 2013-10-10

**Authors:** Florence Gabriel, Frédéric Coché, Dénes Szucs, Vincent Carette, Bernard Rey, Alain Content

**Affiliations:** ^1^Department of Experimental Psychology, Centre for Neuroscience in EducationUniversity of Cambridge, UK; ^2^Laboratoire Cognition, Langage et Développement, Centre de Recherche Cognition et Neurosciences, Université Libre de Bruxelles (ULB)Bruxelles, Belgium; ^3^Service des Sciences de l'Education, Faculté des Sciences Psychologiques et de l'Education, Université Libre de Bruxelles (ULB)Bruxelles, Belgium

**Keywords:** fractions, equivalence, part-whole, proportion, arithmetic operations, fraction subcontructs

## Abstract

Fractions are well known to be difficult to learn. Various hypotheses have been proposed in order to explain those difficulties: fractions can denote different concepts; their understanding requires a conceptual reorganization with regard to natural numbers; and using fractions involves the articulation of conceptual knowledge with complex manipulation of procedures. In order to encompass the major aspects of knowledge about fractions, we propose to distinguish between conceptual and procedural knowledge. We designed a test aimed at assessing the main components of fraction knowledge. The test was carried out by fourth-, fifth- and sixth-graders from the French Community of Belgium. The results showed large differences between categories. Pupils seemed to master the part-whole concept, whereas numbers and operations posed problems. Moreover, pupils seemed to apply procedures they do not fully understand. Our results offer further directions to explain why fractions are amongst the most difficult mathematical topics in primary education. This study offers a number of recommendations on how to teach fractions.

## Introduction

As the joke goes, “three out of two people have trouble with fractions.” Fractions have been known from ancient civilizations until current times, but they still pose major problems when learning mathematics. Babylonian civilization and Egyptians of 4000 years ago already worked with fractions. The processing of fractions is part of our everyday life and is used in situations such as the estimation of rebates, following a recipe or reading a map. Moreover, fractions play a key role in mathematics, since they are involved in probabilistic, proportional and algebraic reasoning. Then why is it so hard for pupils to learn and represent fractions? Fractions have been used for centuries and are manipulated in a great variety of everyday life situations and in mathematics, and yet they are hard for students to grasp and master. In this article, we will try to shed light on children's difficulties when they learn fractions.

Fractions are well-known to constitute a stumbling block for primary school children (Behr et al., 1983; Moss and Case, 1999; Grégoire and Meert, 2005; Charalambous and Pitta-Pantazi, 2007). Understanding difficulties in learning fractions seems absolutely crucial as they can lead to mathematics anxiety, and affect opportunities for further engagement in mathematics and science. Various hypotheses have been proposed in order to explain those difficulties. In this research, we used a theoretical framework based on psychological and educational theories to define problems encountered by pupils when they learn fractions. We tested 4^th^, 5^th^, and 6^th^-graders in order to identify children's difficulties more precisely.

### Different obstacles in learning fraction

#### Whole number bias

Fractions are rational numbers. A rational number can be defined as a number expressed by the quotient a/b of integers, where the denominator, b, is non-zero. According to a recent theory of numerical development, children who have not yet learned fractions generally believe that the properties of whole numbers are the same for all numbers (Siegler et al., 2011). Indeed, one of the main difficulties when learning fractions comes from the use of natural number properties to make inferences on rational numbers, what Ni and Zhou (2005) called the “whole numbers bias.” This bias leads to difficulties conceptualizing whole numbers as decomposable units.

From a mathematical viewpoint, there are fundamental differences between those two types of numbers. Firstly, rational numbers are a densely ordered set, whereas whole numbers form a discrete set. Between two rational numbers, there is an infinity of other rational numbers, while between two natural numbers, there is no other natural number (Vamvakoussi and Vosniadou, 2004). Secondly, another feature of rational numbers is the possibility to write them from an infinity of fractions. This corresponds to the notion of equivalent fractions. Thirdly, faction symbols are a/b types. Pupils often process numerator and denominator as two separate whole numbers (Pitkethly and Hunting, 1996). They apply procedures that can only be used with whole numbers (Nunes and Bryant, 1996). Consequently, typical errors appear in addition or subtraction tasks (e.g., 1/4 + 1/2 = 2/6), and also in fraction comparison (e.g., 1/5 >1/3). In this case, pupils' reasoning can be resumed as follows: if the number is larger, then the magnitude it represents is larger. But when we think about fractions, a larger denominator does not mean a larger magnitude, but a smaller one. Another difficulty appears in multiplication tasks. Multiplying natural numbers always lead to a larger answer, but it is not the case with fractions (e.g., 8 × 1/4 = 2).

The inappropriate generalization of the knowledge about natural numbers is even more resistant as it is widely anterior to the one about rational numbers (Vamvakoussi and Vosniadou, 2004). In order to overcome these mistakes, it would seem necessary for students to perform a conceptual reorganisation which integrates rational numbers as a new category of numbers, with their own rules and functioning (Stafylidou and Vosniadou, 2004). Furthermore, even in adults, knowledge about natural numbers is often preponderant when processing fractions (Bonato et al., 2007; Kallai and Tzelgov, 2009).

#### Different meanings of fractions

Another major difficulty comes from the multifaceted notion of fractions (Kieren, 1993; Brousseau et al., 2004; Grégoire and Meert, 2005). Kieren (1976) was the first to separate fractions into four interrelated categories: ratio; operator; quotient; and measure. The ratio category expresses the notion of a comparison between two quantities, for example when there are three boys for every four girls in a group. So in this case, the ratio of boys to girls is 3:4; the boys representing 3/7 of the group and the girls 4/7 of the group. In the operator category, fractions are considered as functions applied to objects, numbers or sets (Behr et al., 1983). The fraction operator can enlarge or shrink a quantity to a new value. For example, finding 3/4 of a number can be a function where the operation is multiply by 3 divided by 4, or divided by 4 and then multiply by 3. The quotient category refers to the result of a division. For example, the fraction 3/4 may be considered as a quotient, 3/4. In the measure category, fractions are associated with two interrelated notions. Firstly, they are considered as numbers, which convey how big the fractions are. Secondly, they are associated with the measure of an interval. According to Kieren (1976), the part-whole notion of fractions is implicated in these four categories. That is the reason why he did not describe it as a fifth category.

Thereafter, Behr et al. (1983) proposed a theoretical model linking the different categories of fractions. They recommend considering part-whole as an additional category. They also associated partitioning to the part-whole notion. The part-whole category can then be defined as a situation in which a continuous quantity is partitioned into equal size (e.g., dividing a cake into equal parts), and partitioning would be the same with a set of discrete objects (e.g., distributing the same amount of sweets among a group of children).

Other models have been proposed to describe the multiple meanings of fractions (Brissiaud, 1998; Rouche, 1998; Mamede et al., 2005). These models partly overlap, but are not entirely equivalent. For instance, Mamede et al. (2005) present four types of fraction use: quantifying a part-whole relationship, quantifying a quotient, representing an operator, representing a relation between quantities. Meanwhile Grégoire (2008) suggests a different model, in which three categories correspond to three acquisition stages. In the first stage, the fraction is seen as an operator. This notion refers to sharing situations. The second one is the ratio stage which requires a high level of abstraction because one needs to understand that different fractions can represent the same ratio. This is linked to the notion of equivalent fractions. The third and last stage is related to the numerical meaning of fractions. Fractions are here conceived as a new category of numbers, with their own rules and properties.

#### Conceptual and procedural understanding

Another explanation of children's difficulties when learning fractions lies in the articulation between conceptual and procedural knowledge. Previous studies have shown that children would often perform calculations without knowing why (Kerslake, 1986).

Conceptual knowledge can be defined as the explicit or implicit understanding of the principles ruling a domain and the interrelations between the different parts of knowledge in a domain (Rittle-Johnson and Alibali, 1999). It can also be considered as the knowledge of central concepts and principles, and their interrelations in a particular domain (Schneider and Stern, 2005). Conceptual knowledge is thought to be mentally stored in a form of relational representations, such as semantic networks (Hiebert, 1986). It is not tied to a specific problem, but can be generalized to a class of problems (Hiebert, 1986; Schneider and Stern, 2010).

Procedural knowledge can be defined as sequences of actions that are useful to solve problems (Rittle-Johnson and Alibali, 1999). Some authors consider procedural knowledge as the knowledge of symbolic representations, algorithms, and rules (Byrnes and Wasik, 1991). Moreover, procedural knowledge would allow people to solve problems in a quick and effective way as it can easily be automatized (Schneider and Stern, 2010). Therefore, it can be used with few cognitive resources (Schneider and Stern, 2010). However, procedural knowledge is not as flexible as conceptual knowledge and is often bound to specific problem types (Baroody, 2003).

Those two types of knowledge may not evolve in independent ways. Many theories on knowledge acquisition suggest that the generation of procedures is based on conceptual understanding (Halford, 1993; Gelman and Williams, 1997). They argue that children use their conceptual understanding to develop their discovery procedures and adapt acquired procedures to new tasks. According to this approach, children's difficulties when learning about fractions could be interpreted as a use of mathematical symbols without access to their meaning. Procedural knowledge may also influence conceptual understanding. Using procedures would lead to a better conceptual understanding. But few studies support this idea. For instance, Byrnes and Wasik (1991) argue that many children learn the right procedures to multiply fractions, but they never seem to understand the underlying principles. Other authors support a third point of view. Both types of knowledge might progress in an iterative and interactive way (Rittle-Johnson et al., 2001). Conceptual and procedural knowledge might continually and incrementally stimulate each other. Neither would necessarily precede the other.

In mathematics education, teachers seem to focus more on procedural than conceptual knowledge. Children usually learn rote procedures in a repetitive way. This leads to a misunderstanding of mathematical symbols (Byrnes and Wasik, 1991). Consequently many computational errors are due to an impoverished conceptual understanding.

### Our theoretical framework

Taking into account the different theoretical models presented and the issues they arise led us to build our own conceptual framework. In this study exploring the difficulties in learning fractions, two main components were considered: a conceptual component and a procedural component.

The conceptual component was divided in four distinct aspects: proportion, number, measure and part-whole/partition. Part-whole/partition refers to how much of an object (e.g., 1/2 pizza) or a collection (e.g., 1/2 of a bag of sweets) is represented by the fraction symbol (Hecht et al., 2003; Kieren, 1988). Typical tasks used to assess that kind of conceptual knowledge involve shading parts of a figure indicated by a fraction, or the opposite exercise consisting of writing the fraction representing the quantity of a figure that is shaded (Hiebert and Lefevre, 1986; Byrnes and Wasik, 1991; Ni, 2001). Proportion represents the comparison between two quantities. We used comparison of different expressions of the same ratio (e.g., 1/2, 2/4, and 3/?) as it is an adequate way to assess the understanding of proportion. The numerical meaning of fraction refers to the fact that fractions represent rational numbers that can be ordered on a number line (Kieren, 1988). Two relevant tasks were used to assess children's understanding of the numerical meaning of fractions: firstly, number lines on which they are asked to place a fraction, and secondly, indicating which of several given fractions represents the largest quantity (Byrnes and Wasik, 1991; Ni, 2000).

Several variables also held our attention regarding the representation of fractions. Discrete and continuous quantities were used. Children might have greater difficulties to link 2/4 to 2 out 4 for elements of a set than 2/4 of a pie (Ni, 2001). Multiple objects and figures, as well as numerical symbols were introduced to assess the possible interference of certain types of representations (Coquin-Viennot and Camos, 2006). For practical reasons, we did not examine fractions as a measure in this study. This category is closely related to the metric system. The manipulation of fractions as a measure can be made by splitting units of length, area, volume, time, mass, etc. Understanding these measuring situations involves several concepts that are not exclusively related to fractions, such as understanding different unit systems or a good grasp of the decimal position system. Therefore, it is difficult to assess the understanding of this category in isolation from these variables.

Procedural items were those that could be easily solved by applying a procedure that could be implemented without checking for meaning outside that particular procedure. The procedural component involved various operations on fractions, namely the addition and subtraction with or without common denominators, multiplication, and simplification of fractions. Children were given different arithmetical operations to solve as well as simplification exercises.

### Research questions

The main aim of this study was to provide empirical data that could explain difficulties encountered by children when they learn fractions. Our first objective was to analyse the mathematics curriculum of the French Community of Belgium, where this study was conducted. Our second objective was to understand the nature of pupils' difficulties through different categories.

We addressed several research questions regarding children's difficulties when learning fraction. First, we wanted to define more precisely the difficulties encountered by primary school children. Second, one of the goals of this study was to clarify the relationship between conceptual and procedural knowledge of fractions. Does conceptual knowledge of fractions influence procedural knowledge? Or is procedural knowledge sufficient to understand fractions? Our hypothesis is that children's difficulties come from a lack of conceptual understanding of fractions. Their errors would come from the application of routine procedures, but they do not understand the various underlying concepts.

Conceptual knowledge of fractions was assessed through tests about the different meanings of fractions (part-whole, proportion, number), and the different representations of fractions (e.g., association between figural, numeral, and verbal representations). Procedural knowledge about fractions was evaluated through operations on fractions and simplification tasks.

## Methods

### Participants

The test was administered to eight Grade 4 classes (mean age: 9 years 11 months old), eight Grade 5 (mean age: 11 years 1 month old) classes and eight Grade 6 classes (mean age: 12 years old) from five different schools, representing a total sample of 439 participants (214 girls and 225 boys). The choice of these grades was deliberate, as fraction learning usually starts from Grade 4 in the French Community of Belgium where the study was conducted. Informed consent was obtained from parents and the director of every school, as well as from the 24 teachers involved in this research. Assent from children was obtained at the onset of both testing sessions.

### The setting of the study

We analyzed 21 mathematics textbooks recognized by the Education Department of the French Community of Belgium. Fraction concepts used in mathematics textbooks in Grade 4–6 were listed. The goal was to analyse the progression of fraction learning proposed by those textbooks. The most striking observation was that there was a great variety of ways to introduce fractions. In most textbooks, the part-whole concept was considered as the starting point, but in some cases, the measure concept was introduced first. Every concept described in our theoretical framework was represented in the textbooks, but the number of exercises concerning each one of them varied greatly.

We also examined the official mathematics program of the French Community of Belgium. The program presents, in a structured way, the basic skills for the first 8 years of compulsory education, and the skills pupils have to master by the end of each stage (Ministère de la Communauté française, 1999). Fractions were divided into two different categories, Numbers and Quantities. Any requirement at the end of primary school (Grade 6) is briefly reviewed in this section. In the Number category, pupils should be able count, enumerate and classify fractions as well as decimal numbers. They should also be able to calculate, identify and solve operations involving fractions and decimal numbers. In the Quantities category, children are supposed to operate and fractionate different quantities in order to compare them. They should be able to add up and subtract two fractions as well as calculating percentages. The program also mentioned their ability to solve proportionality problems.

The official program offers a list of what pupils should know about fractions in primary school. But what did not appear clearly was a logical progression between all the meanings of fractions. For example, how and when should equivalent fractions be introduced? There was not a clear development for teaching fraction. This situation may be risky as teachers might present fractions as a succession of different independent activities with no real underlying logical progression.

In order to complete the information found in the textbooks, we analyzed pedagogical practices about the way teachers introduce and teach fractions. This investigation revealed the great variety of ways to teach fractions. Our analysis was based on different sources. Firstly, we asked the 24 teachers involved in this study to give us a list of all the activities about fractions conducted in their classrooms. Secondly, teachers gave us a sample of their lessons on fractions as well as pupils notebooks. Thirdly, we made informal observations during the tests.

In Grade 4, pupils learn how to read and represent the value of a fraction. They start placing fractions on a graduated number line. They learn how to simplify fractions (i.e., introduction to equivalent fractions). They learn how to add and subtract of fractions with small and common denominators. In Grade 5, children learn more about fractions as numbers and how they represent quantities. Pupils are trained to convert fractions into decimal numbers and vice versa. They use addition and subtraction of fractions with different denominators. Improper fractions are introduced. In Grade 6, multiplication of fractions is introduced.

Our analysis highlighted the fact that teachers are more inclined to use procedures than what is recommended by the official program. The different conceptual meanings are presented successively without any logical progression. The order in which they are introduced depends on the teacher and on the textbook used by the teacher. Furthermore, fractions seem isolated from mathematics lessons and are taught like a separate topic.

### Test

A test was designed to answer our research questions. Its construction has been guided by our theoretical framework as well as the primary school curriculum in the French Community of Belgium. The test was split into two parts. Part A was made of 19 questions, Part B of 20 questions. There were 1 to 8 items for each question. There were 46 items in Part A and 48 in Part B. Part B was administered one week after Part A. Pupils had 50 min to answer each part.

#### Conceptual knowledge assessment

Conceptual knowledge of fractions was assessed through different categories of questions: part of a whole/partition, proportion and number. Three types of representations have been used: symbolic (e.g., 1/4), verbal (e.g., one-quarter) and figural representations (e.g., a square where the colored part represented 1/4). Discrete and continuous quantities were used.

Multiple variables were taken into account regarding numerical and verbal representations, such as the degree of familiarity, or the parity of the denominator and the numerator. The following variables were controlled regarding figural representations: the equivalence of the parts; the shape of the figure (square, rectangle, triangle …); the size of the figure; and the contiguity of the colored parts of the figure.

***Part-whole/partition***. Part-whole assessment included items for which children had to link fractions to a figural representation. The first question consisted of 6 items for which children were asked to represent a given fraction with a figure (e.g., draw a figure representing 1/7). The items were familiar fractions (1/2 and 3/4), unfamiliar fractions (1/7 and 4/5) and improper fractions (i.e., fractions larger than 1; 3/2 and 7/5). In the second question, pupils were asked to choose a figure representing a given fraction (e.g., choose figures representing 1/4, see Appendix). In the third question, they were asked to shade a certain portion of a figure. There were four items for this question. In the first two items, children were asked to shade 3/4 of a square or a rectangle. In the next two items, they were asked to shade 4/5 of a pentagon or a square.

**Figure 1:**
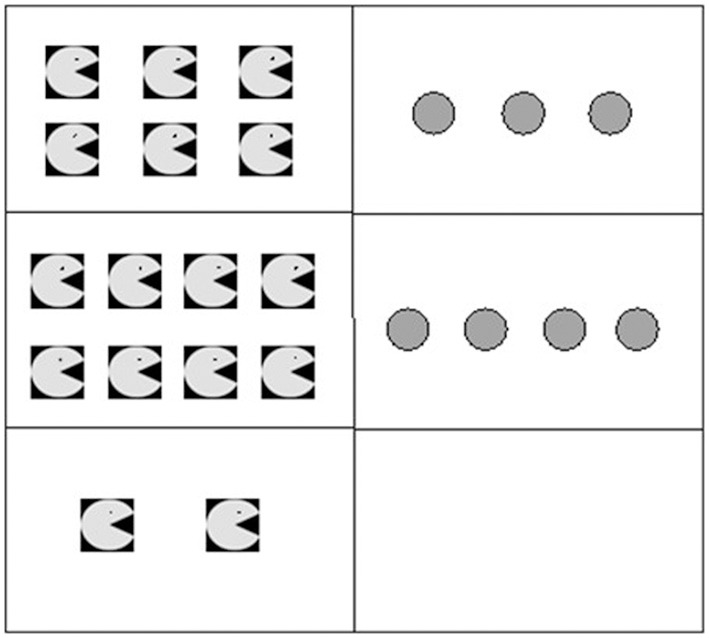
**Example of a figural proportion item**.

***Proportion***. For questions about proportion, children were asked to compare quantities based on the rule of three. Five quantities were given in a table and they had to give the sixth quantity. There were verbal representations, such as “3 cakes cost €6, 5 cakes cost €10, 7 cakes cost €?” There were also figural representations. An example of figural representation is given in Figure 1. The contextualization of the items was introduced to make sure that children based their answer on both columns of the tables.

***Numbers***. For the number category, there were four types of questions. The first question was a comparison of fractions. Pupils had to decide which of two fractions represented the larger quantity. There were fractions with the same numerator (e.g., 2/3_2/7), fractions with the same denominator (e.g., 3/8_5/8) and fractions with no common components (e.g., 2/5_1/4). In the second question, pupils were asked put fractions in ascending order. This question also involved improper fractions and natural numbers. The given numbers were the following: 3/4, 1/2, 8/4, and 1. The third question involved finding a fraction between two given fractions (e.g., find a fraction between 2/7 and 5/7). Fractions with common denominators, common numerators, and no common components were included. For the fourth question, pupils were asked to place a fraction or the unit on a graduated number line (e.g., given 0 and 1/4, place 3/4 on the number line). The given references were always 0 and another fraction.

#### Procedural knowledge assessment

We assessed the following procedures: addition and subtraction with or without the same denominator; multiplication of fractions; multiplication of a fraction by an integer; and simplification of fractions. Those procedures were assessed with typical questions such as 1/2 + 1/4 = ?. Division of fractions was not included as it is not part of the official curriculum.

## Results

### General results

Descriptive statistics are reported for each category of fractions (part-whole, proportion, numbers, operations, and simplification). Mean scores and standard deviations are always expressed in percentage. As can be seen in Table 1, children performed better for questions about proportion and part-whole than for questions about the other categories. There were still major difficulties in Grade 6 for the part-whole category. Indeed, even in Grade 6, the percentage of correct responses was still far from ceiling performance. Children were capable of resolving questions on proportional reasoning from Grade 4. The main observed errors were linked to additive reasoning. Children got the lower scores in Grade 4 for arithmetic operations. This was not surprising as learning about operations on fractions usually start in Grade 5.

**Table 1:** **Mean percentage of correct responses and standard deviation for each category in Grade 4–6**.

	**Part-whole**	**Proportion**	**Number**	**Operations**	**Simplification**
Grade 4	65 ± 16	69 ± 28	47 ± 19	22 ± 18	26 ± 6
Grade 5	72 ± 13	78 ± 26	52 ± 18	37 ± 28	61 ± 9
Grade 6	77 ± 15	85 ± 22	63 ± 20	53 ± 27	71 ± 10

A correlation analysis was run to assess the relations between conceptual (part of a whole, proportion and numbers) and procedural categories (operations and simplification). The correlation analysis revealed that conceptual categories correlated significantly with each other (see Table 2). They also correlated positively with procedural categories.

**Table 2:** **Correlations between conceptual items and procedural items**.

	**Part-whole**	**Proportion**	**Number**	**Operations**	**Simplification**
Part-whole	1				
Proportion	0.348^**^	1			
Numbers	0.382^**^	0.359^**^	1		
Operations	0.383^**^	0.307^**^	0.460^**^	1	
Simplification	0.305^**^	0.386^**^	0.281^**^	0.387^**^	1

** Significant at p < 0.01.

**Figure 2:**
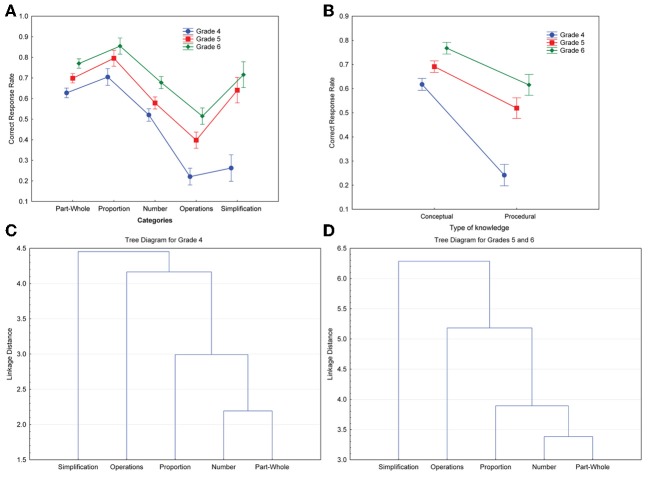
**The top two panels show the interaction between grade and correct response rates for each category (A), and between grade and each type of knowledge (B).** Vertical bars denote 95% confidence intervals. The bottom two panels show dendrograms depicting the results of a single linkage hierarchical clustering of each category based on Euclidian distances for Grade 4 **(C)** and Grades 5 and 6 **(D)**.

We ran an ANOVA for repeated measures with category as a within-subjects factor (part-whole; proportion; number; operations; simplification) and grade as a between-subjects factor. There was a significant grade effect, *F*_(2, 437)_ = 71.53, *p* < 0.001, η^2^_*p*_ = 0.25. There was also a main effect of category, *F*_(4, 1744)_ = 242.64, *p* < 0.001, η^2^_*p*_ = 0.36, and a significant grade x category interaction, *F*_(8, 1744)_ = 19.85, *p* < 0.001, η^2^_*p*_ = 0.08 (see Figure 2A). Tukey *post-hoc* tests showed that accuracy for operations and simplification was poorer in Grade 4 than in Grades 5 and 6 (*p* < 0.001).

We ran another ANOVA for repeated measures on the type of knowledge (conceptual and procedural) with grade as a between-subjects factor. There was a significant effect of grade, *F*_(2, 437)_ = 75.23, *p* < 0.001, η^2^_*p*_ = 0.26. There was also a significant effect of the type of knowledge, *F*_(1, 438)_ = 459.5, *p* < 0.001, η^2^_*p*_ = 0.51, and a significant grade x type of knowledge interaction, *F*_(2, 437)_ = 242.64, *p* < 0.001, η^2^_*p*_ = 0.36 (see Figure 2B). Tukey *post-hoc* test was used to determine significant differences between grade mean values for each type of knowledge, revealing that performance was poorer for procedural knowledge in Grade 4 than in Grades 5 and 6 (*p* < 0.001).

We also ran cluster analyses to ensure that our categories reflected conceptual and procedural knowledge. Since two patterns appeared in the results, we ran two separate cluster analyses: one analysis for Grade 4 and one analysis for Grades 5 and 6. We ran neighbor-joining analyses (single linkage method) to see if our categories formed natural clusters that could be labeled according to a type of knowledge. These analyses provide a tree-structured graph (i.e., dendrogram) that is used to visualize the results of hierarchical clustering calculations. The dendrogram indicates at what level of similarity any two clusters were joined. It was constructed using neighbor-joining algorithm based on Euclidian distances. Both for Grade 4 and for Grades 5 and 6, the dendrograms clustered the categories into two distinct groups that correspond to our two types of knowledge, i.e., conceptual and procedural (see Figures 2C,D). Part-whole, number and proportion were the most similar and correspond to our conceptual categories, whereas operations and simplification can be combined in a different cluster, that is our procedural categories.

### Part-whole/partition

#### Draw a representation for each given fraction

Table 3 shows mean scores and standard deviation for the first question related to the part- whole/partition meaning of fractions. Different variables were involved in this question. Firstly, an ANOVA with the type of fraction as within-subject factor (2 levels: proper fraction vs. improper fraction) was run. Performance was worse for improper fractions than for proper fractions, *F*_(1, 438)_ = 2039.2, *p* < 0.001, η^2^_*p*_ = 0.90. Secondly, familiar (1/2, 3/4) and unfamiliar fractions (1/7, 4/5) were compared in another ANOVA. Performance for familiar fractions was significantly better than for unfamiliar fractions, *F*_(1, 438)_ = 2406.9, *p* < 0.001, η^2^_*p*_ = 0.92.

**Table 3:** **Mean percentage and standard deviation for the question: Draw a representation of the given fraction**.

**Items**	**Grade 4**	**Grade 5**	**Grade 6**
1/2	84 ± 4	95 ± 14	98 ± 10
1/7	67 ± 7	83 ± 5	89 ±2
3/4	75 ± 8	87 ± 4	89 ± 3
4/5	67 ± 5	77 ± 3	90 ± 3
7/5	14 ± 7	20 ± 9	35 ± 9
3/2	23 ± 7	23 ± 11	41 ± 10

**Figure 3:**
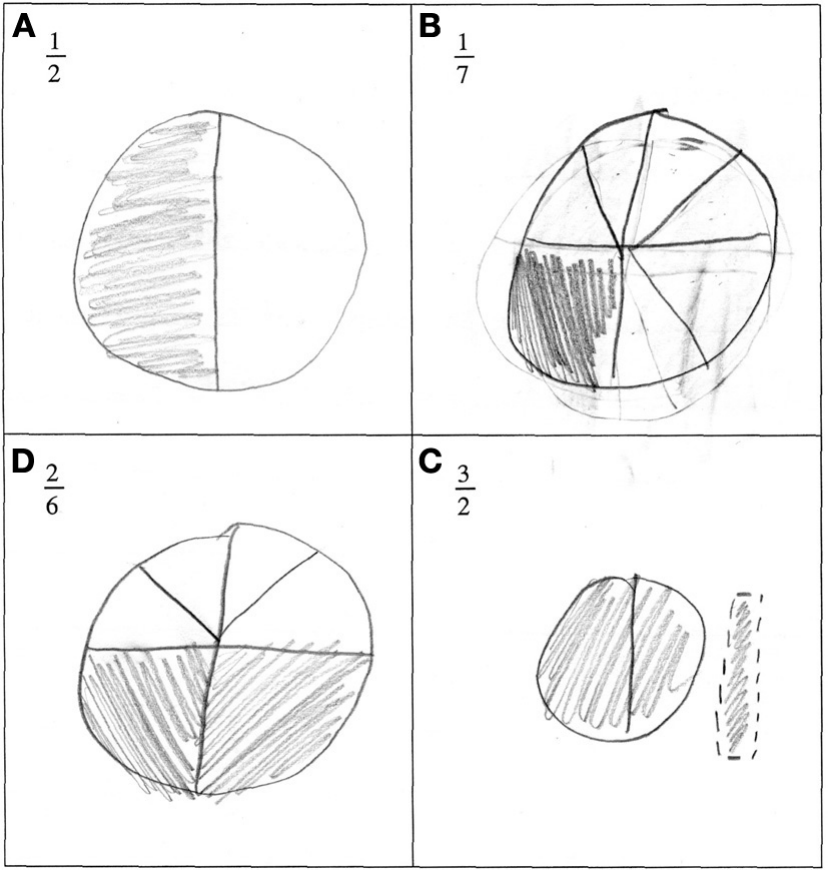
**Illustration of the most common answer when pupils were asked to draw a representation of a given fraction.** 90% of them drew continuous quantities such as a circle or a rectangle. In this particular example, only 1/2 was represented correctly **(A)**. Parts of the drawings were unequal for 1/7 and 2/6 (**B** and **C**). Different shapes were used for 3/2 **(D)**.

Despite potential graphic difficulties, pupils mostly divided a common continuous shape (circle or square, see Figure 3). 90% of pupils represented continuous quantities.

#### Select the figures representing 1/4

In this task, pupils had to choose figures representing the quantity 1/4 (see Appendix). Mean percentage of correct responses were high in every grade (Mean = 92% ± 6%). But when figures were representing 2/8, we observed a dramatic drop of performance: 24 ± 6% in Grade 4, 29 ± 8% in Grade 5 and 59 ± 9% in Grade 6. There was a significant difference between continuous and discrete quantities, *F*_(1, 438)_ = 2308.1, *p* < 0.001, η^2^_*p*_ = 0.91. Performance was better for continuous quantities.

#### Shade a certain fraction of a figure

In this task, pupils had to shade 3/4 or 4/5 of a given figure. Mean scores per grade are given in Table 4. Mean scores for 3/4 (Mean = 83 ± 2%) were higher than for 4/5 (Mean = 65 ± 4%). An ANOVA with familiarity as a within-subject factor showed a significant difference between 3/4 and 4/5, *F*_(1, 438)_ = 3156.6, *p* < 0.001, η ^2^_*p*_ = 0.93.

**Table 4:** **Mean scores and standard deviation for each item in which pupils had to shade 3/4 or 4/5 of a given figure**.

**Figure**	**Fraction**	**Grade 4**	**Grade 5**	**Grade 6**
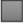	3/4	89 ± 2	88 ± 2	92 ± 1
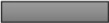	3/4	58 ± 5	82 ± 2	86 ± 1
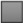	4/5	66 ± 4	62 ± 5	86 ± 2
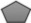	4/5	55 ± 5	53 ± 5	69 ± 2

### Proportion

As seen in Table 1, performance for proportion items was better than in other categories. However, 10% of the answers given by 4^th^-graders were based on additive reasoning. This percentage dropped to 5% in Grade 5 and 2.6% in Grade 6. This type of error was more present for numerical items (Grade 4 = 9%; Grade 5 = 7%; Grade 6 = 3%) than for figural items (Grade 4 = 2%; Grade 5 = 2%; Grade 6 = 1%). A single-factor ANOVA was run and showed no significant difference between numerical and figural items, *F*_(1, 438)_ = 0.6, *p* = 0.8.

### Number

#### Place a given fraction on a number line

Percentage of correct responses showed a clear difference between three groups of items. In the first group of items, there were 3 number lines for which pupils only had to count the number of graduations corresponding to numerators to succeed (e.g., knowing 0 and 5/9 on the fifth graduation, place 2/9). For these items, they could only process the numerator and ignore the denominator. Mean percentage of correct responses for these items was 89 ± 6%. In the second group of items, there were two number lines on which pupils had to place 1 (e.g., knowing 0 and 1/5 on the first graduation, place 1). The mean score for this group of items was the following: Mean = 40 ± 22%. The third group of items involved equivalent fractions (e.g., knowing 0 and 1/6 on the second graduation, place 2/3). The mean score for these items was the following: Mean = 31 ± 24%. An ANOVA with the group of items as a within-subject factor showed a significant difference between the first group of items compared to unit items and items involving equivalent fractions, *F*_(2, 437)_ = 2942.6, *p* < 0. 001, η^2^_*p*_ = 0.95. Tukey *post-hoc* tests showed that the first group of items was higher than unit items (*p* < 0.001) and equivalent fractions items (*p* < 0.001).

Error analysis showed that when asked to place 1 on a number line, pupils had a tendency to place it at the beginning (12% of given responses) or at the end of the line (43% of given responses).

#### Put these fractions in ascending order

Children were asked to sort the following numbers in ascending order: 3/4, 1/2, 8/4, and 1. 55% of 4th-graders placed 1 at the end of the sequence, after 8/4. Furthermore, 22% of 4-graders placed 1 at the beginning of the sequence, before 1/2 and 3/4. This error rate decreased in grades 5 and 6, but 30% of 6th-graders still put 1 at the end of the sequence. These errors are consistent with the errors observed in the number line task. Children struggled with the relation between fractions and the unit.

#### Comparison of fractions

Pupils had to choose which of two fractions was larger. There were three types of items: same denominators (Mean = 83 ± 2%); same numerators (Mean = 56 ± 2%); and no common components (Mean = 65 ± 2%). An ANOVA on the type of fraction (3 levels: same denominators; same numerators; and no common components) revealed significant differences between types, *F*_(2, 437)_ = 1346.4, *p* < 0.001, η^2^_*p*_ = 0.90. Tukey *post-hoc* tests showed that scores for fractions with common denominators were higher than for fractions with common numerators (*p* < 0.001) and fractions with no common components (*p* < 0.001).

### Operations

Performance for addition and subtraction with same denominators was better than for addition and subtraction with different denominators (see Table 5). This is not surprising as addition and subtraction with different denominators are not yet part of the program in Grade 4. But the procedure to find the lowest common denominator seems to pose problems in Grade 5 and 6. The most common error was based on the natural number bias, that is, adding or subtracting numerators and denominators as if there were natural numbers (e.g., = 1/3 + 1/4 = 2/7). 62% of 4th-graders made this mistake for addition and subtraction with different denominators, and this percentage still reached 22% in Grade 6. Surprisingly, performance for multiplication of fractions was better in Grade 4 than in Grade 5. An ANOVA showed significant differences on the types of operations, *F*_(2, 437)_ = 135.5, *p* < 0.001, η^2^_*p*_ = 0.45. Tukey *post-hoc* tests showed that performance was better for addition and subtraction with common denominators than for addition and subtraction with different denominators and multiplication (*p* < 0.001).

**Table 5:** **Mean percentage of correct responses and standard deviation for each type of operations in Grade 4–6**.

	**Addition and subtraction/**	**Addition and subtraction/**	**Multiplication: fraction**	**Multiplication: fraction**
	**same denominators**	**different denominators**	**× integer**	**× fraction**
Grade 4	37 ± 9	1 ± 1	18 ± 7	39 ± 6
Grade 5	51 ± 7	25 ± 8	28 ± 5	36 ± 5
Grade 6	72 ± 7	33 ± 9	43 ± 5	54 ± 4

### Simplification

As can be seen in Table 6, performance in the simplification task was better for fractions that could be divided by 2 (e.g., 4/8) than for fractions that could be divided by 3 (e.g., 15/9), *F*_(1, 438)_ = 384.4, *p* < 0.001, η^2^_*p*_ = 0.64. There was no significant difference between simplification of proper and improper fractions, fractions, *F*_(1, 438)_ = 1.76, *p* = 0.19.

**Table 6:** **Mean percentage of correct responses and standard deviation for the simplification task in each grade**.

	**Grade 4**	**Grade 5**	**Grade 6**
4/10	36 ± 8	72 ± 11	78 ± 10
9/12	20 ± 6	54 ± 9	62 ± 11
15/9	19 ± 6	56 ± 9	71 ± 11
16/4	30 ± 6	63 ± 10	74 ± 9

## Discussion

In this study, we investigated the difficulties encountered by primary school children when learning fractions. One of the main goals of this study was to clarify the relationships between conceptual and procedural understanding of fractions. In order to do so, a test was administered in Grade 4–6 in classes of the French Community of Belgium. The test was based on the different conceptual meanings of fractions, namely part-whole/partition, number, proportion, as well as on procedural questions involving arithmetical operations and simplification of fractions.

Globally, the results showed large differences between categories. Pupils seemed to master the part-whole concept, whereas numbers and operations posed tremendous problems. Some conceptual meanings, such as numbers, were less used in primary school classes. Part-whole seems to be a concept that is widely used in the classrooms. Indeed, children performed well in the part-whole/partition category. However, they seem to have a stereotypic representation of fractions. Indeed, when they were asked to represent a given fraction, they mostly used a circle or a square, even when drawing collections could have been easier (e.g., 1/7). Moreover, when asked to select a figure representing a certain fraction, they performed better for continuous than discrete quantities. Pupils performed well with proportion items. These results contrast with textbooks and lessons given by teachers. In fact, the connection between proportions and fractions is rarely made in textbooks and formal lessons, even if some aspects of fractions are based upon proportional reasoning (e.g., the rule of three).

In the proportion category, most errors were linked to additive reasoning. For example, when pupils are asked questions such as “3 cakes cost €12, 6 cakes cost €24, 8 cakes cost €?” the most common error would be the answer €36. In this case, children built their answer on only a subset of the given information and they applied additive strategies where multiplicative strategies should be used. Mistakes linked to additive reasoning are commonly reported during early stages of children's understanding of proportional reasoning (Lesh et al., 1988). This kind of mistakes was common in Grade 4, but could still be observed in Grade 6.

Pupils performed poorly in the numerical category. Even if children are trained to deal with number lines from grade 4, results showed major difficulties when they were asked to place a fraction on a graduated number line. They do not seem to have an appropriate representation of the quantities of fractions. Other studies have reported that many pupils experience difficulties when asked to locate a fraction on a number line. Pupils often view the whole number line, irrespective of its magnitude as a single unit instead of a scale (Ni, 2001). When they are asked to place a fraction between 0 and 1, pupils often place fractions disregarding any other reference point or known fractions. Pearn and Stephens (2004) pointed out that the incorrect location of fractions could also be the consequence of a lack of accuracy when dividing segments.

The lack of accuracy in children's mental representations of the magnitude of fractions seems to be confirmed by the weak percentage of correct response for questions involving sorting out a range of fractions in ascending order. Furthermore, mean percentage of correct responses for comparison of fractions were very low for fractions with common numerators and fractions no common components. When fractions share the same denominator (e.g., 2/5_4/5), the global magnitude of fractions is congruent with the magnitude of the numerators (e.g., 4 is larger than 2). In this case, pupils could only compare the numerators in order to choose the larger fraction. When fractions share the same numerator, the global magnitude of fractions is incongruent with the magnitude of denominators. Thus, pupils might not take the incongruity into account and their judgment might have been influenced by the whole number bias (Ni and Zhou, 2005). For fractions with no common components, pupils probably only compared numerators and denominators separately. This strategy led to larger error rates.

Focusing now on operations, children performed well in addition and subtraction of fractions with the same denominator, while performance dropped dramatically in addition and subtraction of fractions with different denominators. The most common errors were dictated by the whole number bias (Ni and Zhou, 2005). For example, when asked 3/4 + 2/5 = ?, the majority of pupils answers 5/9. Surprisingly, results were poorer for items involving the multiplication of an integer by a fraction, than for multiplication of two fractions. In the last case, pupils could successfully apply procedures based on natural numbers knowledge, which would explain higher percentage of correct response. Another surprising result was the better performance in Grade 4 than Grade 5 when children were asked to multiply an integer by a fraction. There might be a contamination of procedures applied to addition and subtraction with different denominators learnt in Grade 5.

Results showed massive familiarity effects in every category. Children performed significantly better on questions including familiar fractions, such as 1/2, 1/4, or 3/4 than on items with less familiar fractions. This could be due to the fact that the magnitude of 1/2 is known better than other fractional magnitudes. We do not know precisely when children start to quantify continuous quantities in informal contexts. Bryant (1974) suggests that children are able to understand part/part relations before part/whole relations. Relations such as “larger than/smaller than” and “equals to” could be the first logical relationships used at the beginning of fraction learning. Spinillo and Bryant (1991) designed experiments to analyse how 4- to 7-year-olds use the concept of “half” in equivalence judgment tasks. Their results suggest that using the concept of half would be the first step in relationships used by children to quantify fractions.

Desli (1999) also investigated the role of half by examining part/whole relationships. 6- to 8-year-olds were told that two parties had been organized and that chocolate bars would be equally distributed among children. They had to judge if they would receive the same amount of chocolate bars in both parties, and if not, in which party they would get more chocolate bars. Children had ceiling performance when they could use half as a reference. In the condition where they could not use half as a reference, only 8-year-olds had performance above chance. Desli (1999) also showed the importance of the concept of half in the construction of fractions quantifications. In a recent study using a fraction-based judgment task, Mazzocco et al. (2013) showed that fractions equivalent to 1/2 were easier to conceptualize. Moreover, children as young as 3 and 4 years old already have a good representation of the half boundary (Singer-Freeman and Goswami, 2001). As children are frequently exposed to 1/2 quite early in life, the familiarity of that quantity might induce a different type of mental representations compared to other less familiar fractions. Pupils might benefit from lessons including a larger pool of fractions. Teaching programs mostly insist on quantities that can be divided by 2. This limited vision of fractions seems to generate difficulties when it comes to generalization. Teachers could diversify the number of fractions used during lessons.

Improper fractions represented another major difficulty for primary school children (Bright et al., 1988; Tzur, 1999). The main difficulty appeared in the test when pupils were asked to graphically represent an improper fraction or when an improper fraction was presented in an ordering task. When pupils were asked to order 1 in a sequence involving fractions, the most common error was to put it at the end of the sequence, even if there was an improper fraction. This could mean that some children cannot imagine fractions can be larger than 1. This is consistent with the results found by Kallai and Tzelgov (2009) who showed that adults have a mental representation of what they called a “generalized fraction.” A “generalized fraction corresponds to an “entity smaller than one” emerging from the common notation of fraction (Kallai and Tzelgov, 2009).

Furthermore, children seem to have a limited conception of the relation between 1 and fractions. Looking at questions on number lines and the ordering task, we observed two different conceptions regarding the number 1. In the first case, 1 was put at the beginning of the sequence. This can be interpreted as 1 being at the beginning of counting sequence. This error is again linked to the whole number bias (Ni and Zhou, 2005). Indeed, pupils based their answer on prior knowledge and the expectation that fractions follow the same rule of counting as whole numbers. In the second case, 1 was placed at the end of the sequence. Children who made this mistake considered fractions as being entities smaller than one.

Equivalent fractions were not understood by the majority of children (Kamii and Clark, 1995; Arnon et al., 2001). For example, performance was poor when they were asked to place 2/3 on a number line when the references were 0 and 1/6. Yet, their score was high for questions involving simplification of fraction. There was a clear dissociation between conceptual and procedural understanding. Children mastered the procedure applied to simplify fractions, but did not seem to understand the underlying concept of equivalent fractions.

To sum up, the test that we designed revealed many weaknesses in understanding fractions in primary school. Teaching practice seems to focus more on procedures than on conceptual understanding of fractions. But our results showed that procedures are not sufficient to carry out operations with fractions for instance. Even if pupils are intensively trained with finding the least common denominators procedure, the percentage of correct responses for addition and subtraction with different denominators remained low. Conceptual understanding is essential to ensure a deep understanding of fractions. In the U.S., it is already been recommend for the teaching of fractions (NCTM, 2000; Fazio and Siegler, 2012), and based on our results, we would suggest this recommendation should also apply for the French Community of Belgium.

We argue that children might benefit from a training based on concrete objects manipulation and explicit learning of rational numbers characteristics. Teaching children concrete activities could help them develop the corresponding abstract concepts (Arnon et al., 2001; Gabriel et al., 2012). For example, most primary school children consider fractions as being entities smaller than one (Behr et al., 1992; Stafylidou and Vosniadou, 2004). Moreover, most of them do not seem to understand equivalent fractions. These particular characteristics constitute the main differences between fractions and natural numbers. Pupils might benefit from more training with concrete objects to realize the necessary conceptual reorganisation and understand the properties of fractions. Another interesting finding of this study is that children performed better with familiar fractions. It could be interesting to introduce a larger variety as well as diversified representations of fractions in lessons. By integrating a larger range of fractions, children might get a more flexible representation of the magnitude of fractions.

Unfortunately, our experiment did not allow us to draw conclusions on how conceptual and procedural knowledge influence each other. Correlation analysis revealed that every conceptual and procedural items were positively correlated with each other. Therefore, links between conceptual and procedural understanding are hard to interpret. This might mean that both types of knowledge are not independent and could be equally important when learning fractions. Both types of knowledge might evolve in an iterative way. Besides, individual differences have been reported in the development of conceptual and procedural knowledge (Hallett et al., 2010; Hecht and Vagi, 2012). Children differ in the use of conceptual and procedural knowledge to solve fraction problems (Hallett et al., 2010). Another reason can account for the difficulties to interpret findings obtained with a hypothetical measure of conceptual and procedural knowledge. The assessment of conceptual knowledge might reflect, to some extent, procedural knowledge and vice versa (Rittle-Johnson and Alibali, 1999). Future investigations are required to shed light on the links between conceptual and procedural knowledge in fraction learning and examine the possible reasons for individual differences.

In conclusion, our results showed that primary school children master the part-whole and proportion categories, but they struggle to understand fractions as numbers. Equivalent and improper fractions are very difficult to grasp, and pupils seem to apply procedures that they do not really understand. This might be linked to teaching practice that allocates more time and exercises only based on procedures.

### Conflict of interest statement

The authors declare that the research was conducted in the absence of any commercial or financial relationships that could be construed as a potential conflict of interest.

## Figures and Tables

**Figure A1:**
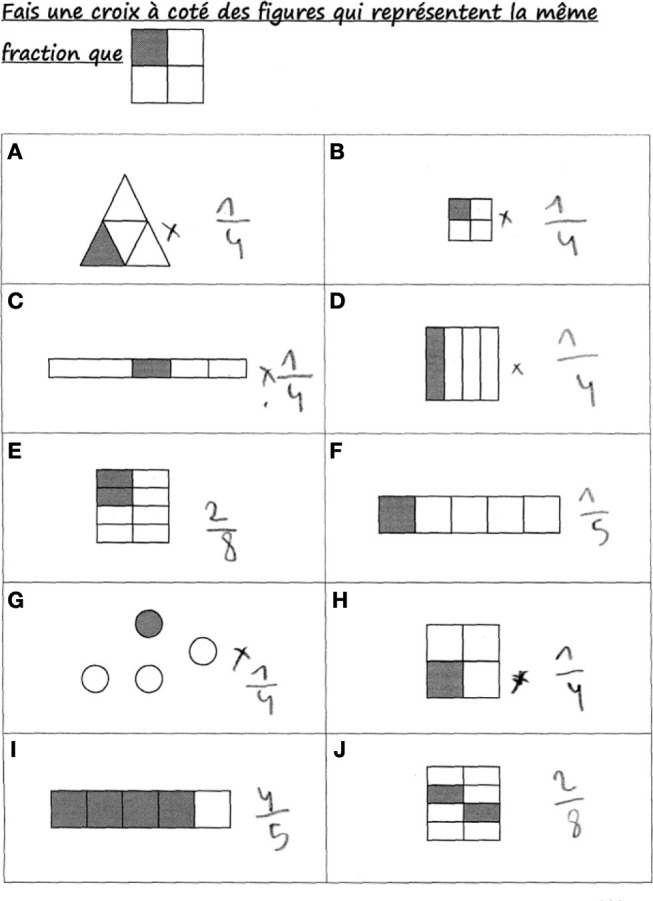
**Part-whole item: select fractions representing the same fraction as 1/4.** To get full marks, children had to select panels **(A,B,D,E,G,H)** and **(J)**. In this particular example, the pupil failed to spot fractions equivalence.

## References

[B1] Arnon I. Nesher P. Nirenburg R. (2001). Where do fractions encounter their equivalents? Can this encounter take place in elementary-school? Int. J. Comput. Math. Learn. 6, 167–214 10.1023/A:1017998922475

[B2] Baroody A. J. (2003). “The development of adaptive expertise and flexibility: the integration of conceptual and procedural knowledge,” in The Development of Arithmetic Conceptsand Skills: Constructing Adaptive ExpertiseI, eds Baroody A. J. Dowker A. (Mahwah, NJ: Erlbaum), 1–33

[B3] Behr M. J. Harel G. Post T. Lesh R. (1992). “Rational number, ratio, and proportion,” in Handbook of Research on Mathematics Teaching and Learning, ed Grouws D. A. (New York, NY: Macmillan), 296–333

[B4] Behr M. J. Lesh R. Post T. R. Silver E. A. (1983). “Rational numbers concepts,” in Acquisition of Mathematics Concepts and Processes, eds Lesh R. Landau M. (New York, NY: Academic Press), 91–125

[B5] Bonato M. Fabbri S. Umiltà C. Zorzi M. (2007). The mental representation of numerical fractions: real or integer? J. Exp. Psychol. Hum. Percept. Perform. 33, 1410–1419 10.1037/0096-1523.33.6.1410 18085953

[B6] Bright G. Behr M. Post T. Wachsmuth I. (1988). Identifying fractions on number lines, J. Res. Math. Educ. 19, 215–232 10.2307/749066

[B7] Brissiaud R. (1998). “Les fractions et les décimaux au CM1. Une nouvelle approche,” in Actes du XXVème Colloque des Formateurs et Professeurs de Mathématiques chargés de la Formation des Maîtres, (IREM de Brest), 147–171

[B44] Brousseau G. Brousseau N. Warfield V. (2004). Rationals and decimals as required in the school curriculum. Part 1: rationals as measurements. J. Math. Behav. 23, 1–20 10.1016/j.jmathb.2003.12.001

[B49] Bryant P. (1974). Perception and Understanding in Young Children: An Experimental Approach, Vol. 588 London: Methuen

[B8] Byrnes J. P. Wasik B. A. (1991). Role of conceptual knowledge in mathematical procedural learning. Dev. Psychol. 27, 777–786 10.1037/0012-1649.27.5.777

[B9] Charalambous C. Pitta-Pantazi D. (2007). Drawing on a theoritical model to study students' understandings of fractions. Educ. Stud. Math. 64, 293–316 10.1007/s10649-006-9036-2

[B10] Coquin-Viennot D. Camos V. (2006). “Décimaux et fractions,” in La cognition mathématique chez l'enfant, eds Barrouillet P. Camos V. (Marseille: Solal), 145–154

[B51] Desli D. (1999). Children's Understanding of Intensive Quantities. Unpublished PhD thesis. Institute of Education, University of London

[B11] Fazio L. Siegler R. (2012). Teaching fractions. Educ. Pract. Ser. 22, 1–28

[B12] Gabriel F. Coché F. Szucs D. Carette V. Rey B. Content A. (2012). Developing children's understanding of fractions: an intervention study. Mind Brain Educ. 6, 137–146 10.1111/j.1751-228X.2012.01149.x

[B13] Gelman R. Williams E. M. (1997). “Enabling constraints for cognitive development and learning: domain specificity and epigenesist,” in Cognitive Development, Handbook of Child Psychology, 5th Edn, eds Kuhn D. Siegler R. (New York, NY: Wiley), 575–630

[B14] Grégoire J. (2008). Aux Sources des Difficultés de L'apprentissage des Fractions. Brussels: Seminar given at, Université Libre de Bruxelles.

[B15] Grégoire J. Meert G. (2005) “L'apprentissage des nombres rationnels et ses obstacles,” in Les Troubles Du Calcul, ed Noël M.-P. (Marseille: Solal), 223–251

[B16] Halford G. S. (1993). Children's Understanding: The Development of Mental Models. Hillsdale, NJ: Erlbaum

[B17] Hallett D. Nunes T. Bryant P. (2010). Individual differences in conceptual and procedural knowledge when learning fractions. J. Educ. Psychol. 102, 395–406 10.1037/a0017486

[B54] Hecht S. A. Vagi K. J. (2012). Patterns of strengths and weaknesses in children's knowledge about fractions. J. Exp. Child Psychol. 111, 212–229 10.1016/j.jecp.2011.08.012 21945345 PMC3225691

[B18] Hecht S. A. Close L. Santisi M. (2003). Sources of individual differences in fraction skills. J. Exp. Child Psychol. 86, 277–302 10.1016/j.jecp.2003.08.003 14623213

[B19] Hiebert J. (ed.). (1986). Conceptual and Procedural Knowledge: the Case of Mathematics. Hillsdale, NJ: Erlbaum

[B20] Hiebert J. Lefevre P. (1986). “Conceptual and procedural knowledge in mathematics: an introductory analysis,” in Conceptualand Procedural Knowledge: the Case of Mathematics, ed Hiebert J. (Hillsdale, NJ: Lawrence Erlbaum Associates), 1–27

[B21] Kallai A. Tzelgov J. (2009). A generalized fraction: an entity smaller than one on the mental number line. J. Exp. Psychol. Hum. Percept. Perform. 35, 1845–1864 10.1037/a0016892 19968440

[B22] Kamii C. Clark F. B. (1995). Equivalent fractions: their difficulty and educational implications. J. Math. Behav. 14, 365–378 10.1016/0732-3123(95)90035-7

[B46] Kerslake D. (1986). Fractions: Children's Strategies and Errors. A Report of the Strategies and Errors in Secondary Mathematics Project. England: NFER-NELSON Publishing Company, Ltd.

[B23] Kieren T. E. (1976). “On the mathematical, cognitive, and instructional foundations of rational numbers,” in Number and Measurement: Papers from a Research Workshop, ed Lesh R. (Columbus, OH: ERIC/SMEAC), 101–144

[B24] Kieren T. E. (1988) “Personal knowledge of rational numbers: its intuitive and formal development,” in Research Agenda for Mathematics Education: Number Concepts and Operations in the Middle Grades, Vol. 2, eds Hiebert J. Behr M. (Virginia: Lawrence Erlbaum), 162–181

[B43] Kieren T. E. (1993). “Rational and Fractional Numbers: From Quotient fields to Recursive understanding,” in Rational Numbers: An Integration of Research, eds Carpenter T. P. Fennema E. Romberg T. A. (Hillsdale, NJ: Erlbaum), 49–84

[B25] Lesh R. Post T. Behr M. (1988). “Proportional reasoning,” in Number Concepts and Operations in the Middle Grades, eds Hiebert J. Behr M. (Reston, VA: Lawrence Erlbaum and National Council of Teachers of Mathematics), 93–118

[B45] Mamede E. Nunes T. Bryant P. (2005). “The equivalence and ordering of fractions in part-whole and quotient situations,” in PME CONFERENCE, Vol. 29, 3

[B26] Mazzocco M. M. M. Myers G. F. Lewis K. E. Hanich L. B. Murphy M. M. (2013). Limited knowledge of fraction representations differentiates middle school students with mathematics learning disability (dyscalculia) vs. low mathematics achievement. J. Exp. Child Psychol. 115, 371–387 10.1016/j.jecp.2013.01.005 23587941 PMC4000738

[B27] Ministère de la Communauté française, (1999). Socles de Compétences Enseignement Fondamental et Premier degré de l'enseignement Secondaire. Test de Mathématique, Résultats et Commentaires. Bruxelles: Administration générale de l'Enseignement et de la Recherche scientifique

[B28] Moss J. Case R. (1999). Developing children's understanding of the rational numbers: a new model and an experimental curriculum. J. Res. Math. Educ. 30, 122–147 10.2307/749607

[B29] National Council of Teachers of Mathematics. (2000). Principles and Standards for School Mathematics. Reston, VA: author

[B48] Ni Y. (2000). How valid is it to use number lines to measure children's conceptual knowledge about rational number. Educ. Psychol. 20, 139–152 10.1080/713663716

[B30] Ni Y. (2001). Semantic domains of rational numbers and the acquisition of fraction equivalence. Contemp. Educ. Psycol. 26, 400–417 10.1006/ceps.2000.1072 11414728

[B42] Ni Y. Zhou Y. D. (2005). Teaching and learning fraction and rational numbers: the origins and implications of whole number bias. Educ. Psychologist 40, 27–52 10.1207/s15326985ep4001_3

[B31] Nunes T. Bryant P. (1996). Children Doing Mathematics. Oxford: Blackwell

[B32] Pearn C. Stephens M. (2004). “Why you have to probe to discover what year 8 students really think about fractions,” in Paper Presented at the Mathematics Education for the Third Millennium: Towards 2010, (Townsville).

[B33] Pitkethly A. Hunting R. (1996). A review of recent research in the area of initial fraction concepts. Educ. Stud. Math. 30, 5–38 10.1007/BF001637518678283

[B47] Rittle-Johnson B. Siegler R. S. Alibali M. W. (2001). Developing conceptual understanding and procedural skill in mathematics: an iterative process. J. Educ. Psychol. 93, 346–362 10.1037/0022-0663.93.2.346

[B34] Rittle-Johnson B. Alibali M. W. (1999). Conceptual and procedural knowledge of mathematics: does one lead to the other? J. Educ. Psychol. 91, 175–189 10.1037/0022-0663.91.1.175

[B35] Rouche N. (1998). L'esprit des Sciences. Pourquoi ont-ils Inventé les Fractions? Paris: Ellipses

[B36] Schneider M. Stern E. (2005). “Conceptual and procedural knowledge of a mathematics problem: their measurement and their causal interrelations,” in Paper Presented at the 27th Annual Meeting of the Cognitive Science Society (CSS), (Stresa).

[B37] Schneider M. Stern E. (2010). The developmental relations between conceptual and procedural knowledge: a multimethod approach. Dev. Psychol. 46, 178–192 10.1037/a0016701 20053016

[B38] Siegler R. S. Thompson C. A. Schneider M. (2011). An integrated theory of whole number and fractions development. Cogn. Psychol. 62, 273–296 10.1016/j.cogpsych.2011.03.001 21569877

[B52] Singer-Freeman K. E. Goswami U. (2001). Does half a pizza equal half a box of chocolates?: proportional matching in an analogy task. Cogn. Dev. 16, 811–829 10.1016/S0885-2014(01)00066-1

[B50] Spinillo A. G. Bryant P. (1991). Children's proportional judgments: The importance of “half”. Child Dev. 62, 427–440 10.1111/j.1467-8624.1991.tb01542.x

[B39] Stafylidou S. Vosniadou S. (2004). The development of students' understanding of the numerical value of fractions. Learn. Instr. 14, 503–518 10.1016/j.learninstruc.2004.06.015

[B40] Tzur R. (1999). An integrated study of children's construction of improper fractions and the teacher's role in promoting that learning. J. Res. Math. Educ. 30, 390–416 10.2307/749707

[B41] Vamvakoussi X. Vosniadou S. (2004). Understanding the structure of the set of rational numbers: a conceptual change approach, *Learn.* Instr. 14, 453–467 10.1016/j.learninstruc.2004.06.013

